# Bilateral ectopia lentis with isolated lens coloboma in Marfan syndrome

**DOI:** 10.3205/oc000051

**Published:** 2016-12-05

**Authors:** Sabin Sahu, Reena Yadav, Sharad Gupta, Lila Raj Puri

**Affiliations:** 1 Sagarmatha Choudhary Eye Hospital, Lahan, Nepal

**Keywords:** ectopia lentis, lens coloboma, Marfan syndrome

## Abstract

A rare case of bilateral ectopia lentis with isolated lens coloboma in Marfan syndrome is reported. A 21-year-old female presented with decreased vision in both eyes. Her unaided visual acuity was 20/200 and 20/400 in the right and left eye, respectively, improving to 20/40 with –4.5 DS/–3.0 DC x 10° correction in the right eye and 20/80 with –10.0 DS/–6.5 Dc x10° correction in the left eye. On slit lamp examination under mydriasis, both eyes revealed ectopia lentis with lens coloboma and stretched zonules. Fundus examination revealed pigmentary changes at the fovea. On systemic evaluation, she was diagnosed with Marfan syndrome. She was prescribed a refractive correction in form of a contact lens and kept under observation.

## Introduction

Marfan syndrome is a systemic disease with various ocular abnormalities. The most common ocular abnormality seen is ectopia lentis, occurring in 50–87% of the affected individuals. Other ocular manifestations include microspherophakia, lens opacities, ocular and lens coloboma, flattened cornea and astigmatism, myopia, megalocornea, glaucoma, strabismus, amblyopia, hypoplastic iris and ciliary muscles, retinal degenerations and detachment [1]. Angra et al. [2], Mehrotra et al. [3] and Thapa et al. [4] described the association of lens coloboma with Marfan syndrome. We report a case of bilateral ectopia lentis with isolated lens coloboma in a patient with Ghent proven Marfan syndrome [5]. 

## Case description

A 21-year-old female presented with decreased vision in both eyes (left more than right) noticed for last one year. Her unaided visual acuity was 20/200 and 20/400 in the right and left eye, respectively, improving to 20/40 with –4.5 DS/–3.0 DC x10° correction in the right eye and 20/80 with –10.0 DS/–6.5 Dc x10° correction in the left eye. Intraocular pressures were 14 mmHg and 16 mmHg in the right and left eye, respectively. 

**Figure 1:**
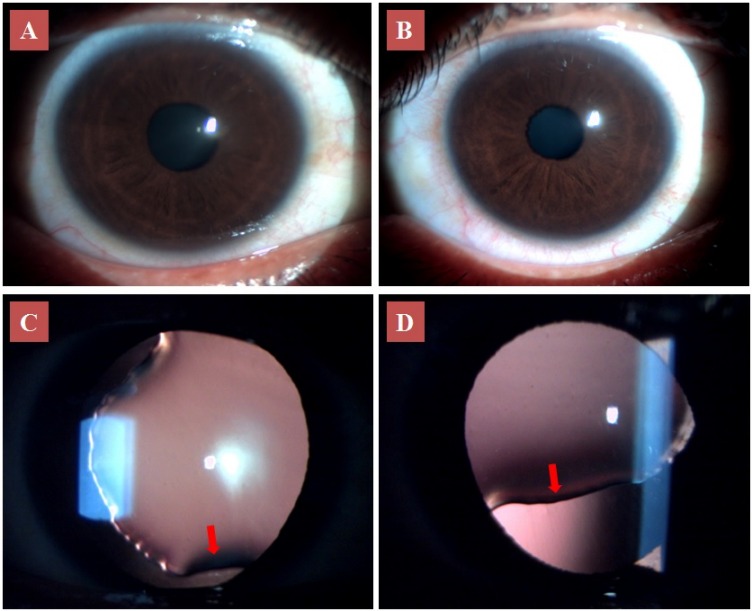
Anterior segment photo of the right (A) and left eye (B) in undilated pupil. Retroillumination view under mydriasis of the right eye (C) showing ectopia lentis with lens coloboma (arrow) and the left eye (D) showing ectopia lentis with lens coloboma and stretched zonules (arrow).

**Figure 2:**
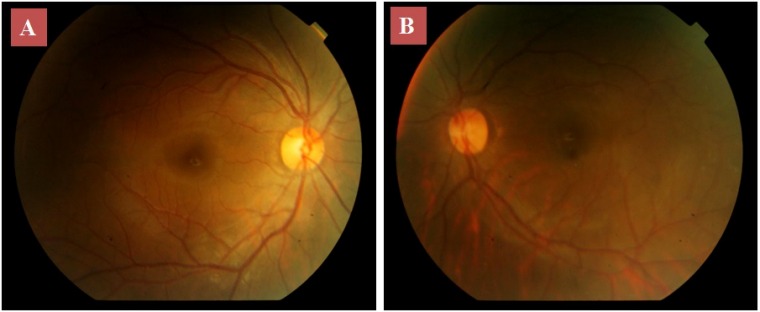
Posterior segment photograph of the right (A) and left (B) eye showing pigmentary changes at the fovea

On slit lamp examination, cornea, iris, anterior chamber and lens appeared normal in undilated pupil in both eyes (Figure 1 A, B (Fig. 1)). However on examination under mydriasis, the right eye revealed ectopia lentis with superonasal subluxation extending from 5 to 12 o’clock with lens coloboma at lens equator (Figure 1 C (Fig. 1)). The left eye revealed ectopia lentis with superonasal subluxation and lens coloboma inferiorly (Figure 1 D (Fig. 1)). The edges of coloboma were notched with present but stretched zonules in these regions. Gonioscopy showed normal angle structures. The fundus examination of both eyes revealed pigmentary changes at the fovea, which could also contribute to a decreased visual acuity (Figure 2 (Fig. 2)). She had no history of trauma or surgery. She did not have any other detected systemic illness. 

Systemic examination revealed a height of 160 cm, with long and thin extremities, a larger arm span than height (arm span / height ratio >1.05) and reduced upper segment / lower segment ratio (<0.85). Arachnodactyly, positive wrist and thumb signs, joint hypermobility, reduced elbow extension, high arched palate, and mild scoliosis were other findings. She had no cardiovascular, pulmonary, renal or central nervous system abnormalities. Family history revealed that her father and one younger brother were of tall stature, suggestive of Marfan syndrome. 

An automated keratometry showed normal corneal curvatures. The axial length of the right eye was 23.62 mm and that of the left eye was 25.09 mm. ECG done was normal.

She was prescribed a refractive correction in form of a contact lens and kept under observation.

## Discussion

Coloboma of lens is characterized by notching at the equator of the lens. It is usually monocular and may or may not be associated with other ocular abnormalities [6]. In the region of lens coloboma the zonules are usually absent or maldeveloped due to an incomplete closure of the optic vesicle or due to persistent remnants of fibrovascular sheath of the lens interfering with the development of the zonules [3]. But similar to the case described by Mehrotra et al. [3], the zonules were present but scanty in the region of lens coloboma in our case as well. A maldevelopment of the zonules was not seen, so the lens coloboma is probably an isolated presentation. 

Marfan syndrome is an autosomal dominant disorder of connective tissue with variable expression. It is characterized by systemic and ocular manifestations due to mutation in the fibrillin gene. Lens coloboma is a less common ocular feature of Marfan syndrome. It is thought to occur secondary to failure of the fetal fissure to completely close, although in Marfan syndrome it may represent secondary filamentary degeneration of zonular fibers and may be accompanied by other ocular coloboma of the eyelid, iris, choroid, or optic disc [1]. Lens coloboma in association with Marfan syndrome has been described by Angra et al. [2], Mehrotra et al. [3] and Thapa et al. [4]. We report a case of bilateral ectopia lentis with isolated lens coloboma in an adult Nepali patient with Marfan syndrome. The patient had a clear lens with good refractive corrections so she was provided optical rehabilitation with a contact lens and kept under regular follow-up.

## Notes

### Competing interests

The authors declare that they have no competing interests.
